# Urinary nephrin as an early biomarker of podocyte dysfunction in essential hypertension: a stage-stratified study from Bukhara, Uzbekistan

**DOI:** 10.3389/fcvm.2026.1878203

**Published:** 2026-07-24

**Authors:** Mukhammad Komil ogli Amonov

**Affiliations:** Department of Faculty and Hospital Therapy, Bukhara State Medical Institute named after Abu Ali ibn Sino, Bukhara, Uzbekistan

**Keywords:** Central Asia, essential hypertension, nephroprotection, podocyte dysfunction, renal functional reserve, TGF-*β*1, urinary nephrin

## Abstract

Essential hypertension is a leading cause of chronic kidney disease, but conventional renal markers detect injury only after substantial nephron loss. Urinary nephrin, a slit-diaphragm protein released during podocyte injury, has been proposed as an early biomarker of hypertensive nephropathy, yet stage-specific data are scarce, particularly from Central Asia. In this single-centre, prospective study, we enrolled 60 adults with essential hypertension stratified by stage (I, II, III; *n* = 20 per group) in Bukhara, Uzbekistan; patients with diabetes, heart failure, primary kidney disease, or prior SARS-CoV-2 infection were excluded. Urinary nephrin, serum cystatin-C, TGF-*β*1, aldosterone, and VEGF-A were measured by ELISA, and renal Doppler ultrasonography and an oral protein-loading test for renal functional reserve (RFR) were performed at baseline and after 6 months of an ACE inhibitor or angiotensin receptor blocker, with eplerenone where indicated. Urinary nephrin rose progressively across stages (91.9 ± 8.3, 124.9 ± 9.3, 164.5 ± 9.7 ng/mL) and was already elevated in stage I despite normal creatinine, cystatin-C, and eGFR. Nephrinuria correlated with systolic blood pressure (r = 0.58), aldosterone (r = 0.54), and microalbuminuria (r = 0.71), and inversely with eGFR (r = −0.74) and RFR (r = −0.76; all *P* < 0.01). After 6 months, urinary nephrin declined by 32%, 31%, and 23% across stages, with parallel reductions in TGF-*β*1, VEGF-A, and aldosterone and partial RFR recovery. Urinary nephrin is elevated while conventional markers remain normal and responds to nephroprotective therapy, supporting its potential as an early biomarker of hypertensive kidney injury.

## 1 Introduction

Essential hypertension is among the most prevalent non-communicable diseases worldwide and a leading driver of cardiovascular morbidity, chronic kidney disease (CKD), and premature death. The World Health Organization estimates that approximately 1.4 billion adults aged 30–79 years have hypertension globally, representing 33% of the population in this age range, with two-thirds living in low- and middle-income countries (1). The 2023 WHO Global Report on Hypertension further documented that only 54% of adults with hypertension are diagnosed, 42% receive treatment, and just 21% achieve adequate control, underscoring the magnitude of the unmet clinical need (2). Cardiovascular disease, of which hypertension is a principal driver, has emerged as a major public health challenge across Central Asia, including Uzbekistan, where post-Soviet transitions, urbanisation, and shifts in lifestyle have all contributed to rising prevalence (3, 4). Recent regional analyses have further highlighted that the management of cardiovascular disease in Central Asia, including the Bukhara region, is complicated by uneven access to specialised diagnostic services, limited availability of modern pharmacotherapy, and persisting urban–rural disparities in care delivery (5). Hypertensive nephropathy is one of the most consequential long-term complications of poorly controlled blood pressure. In developed economies, hypertension is the second leading cause of end-stage kidney disease after diabetes mellitus and accounts for a substantial proportion of patients entering renal replacement therapy programmes (6, 7). The pathological hallmark of hypertensive nephropathy is progressive nephrosclerosis driven by glomerular hypertension, podocyte injury, and tubulointerstitial fibrosis, with epithelial-to-mesenchymal transition increasingly recognised as a unifying mechanism (8). Once these structural changes are established, they are largely irreversible, emphasising the clinical importance of detecting renal damage at a stage when it can still be modified by therapeutic intervention. Current routine screening for renal involvement in hypertensive patients relies on serum creatinine, estimated glomerular filtration rate (eGFR), and urinary albumin excretion. However, each of these markers has well-recognised limitations. Serum creatinine and eGFR remain within the reference range until approximately half of nephron mass has been lost, and the recently introduced 2021 race-free CKD-EPI equation, while improving estimation accuracy, does not overcome this fundamental insensitivity to early disease (9). Microalbuminuria, although recommended for annual screening in hypertensive adults, has been shown to be a relatively late event, with reported sensitivities as low as 41%–45% for detecting incipient hypertensive nephropathy (10, 11). Cystatin-C, although more sensitive than creatinine in early CKD and now incorporated into the KDIGO 2024 CKD guideline, is influenced by non-renal factors including thyroid status, glucocorticoid exposure, adiposity, and systemic inflammation (12, 13). As a result, a substantial proportion of hypertensive patients with ongoing podocyte injury remain undetected by standard nephrology workup until later disease stages, when therapeutic options are constrained.

Podocytes — terminally differentiated visceral epithelial cells lining the outer surface of the glomerular basement membrane — are now recognised as the central target of haemodynamic and humoral injury in hypertensive nephropathy (14). The slit diaphragm bridging adjacent podocyte foot processes is the principal size-selective barrier of the glomerular filtration apparatus, and nephrin is its key structural and signalling protein. Mechanical stretch from elevated intraglomerular pressure, together with activation of the renin-angiotensin-aldosterone system (RAAS) within the podocyte itself, leads to cytoskeletal remodelling, foot-process effacement, and shedding of nephrin into the urinary space (15, 16). Because this process can begin before measurable proteinuria or filtration loss, urinary nephrin has been proposed as a biomarker capable of capturing the earliest stages of glomerular injury (17, 18). Recent advances in quantification methodology, including multiplexed mass-spectrometry assays for nephrin, podocalyxin, and podocin, now permit reliable measurement in urine and have established baseline reference ranges in healthy adults (19, 20). Despite a growing body of experimental evidence supporting this concept, clinical data on urinary nephrin in essential hypertension remain limited and geographically uneven. Most published cohorts originate from high-income countries and focus on diabetic nephropathy, preeclampsia, or established CKD (17, 21). Comparatively little is known about the behaviour of urinary nephrin across the clinical stages of essential hypertension itself, and to our knowledge no published data are available from Central Asia — a region where hypertension is highly prevalent, access to advanced nephrology services is limited, and primary care decisions must often be made without renal biopsy or sophisticated imaging. Beyond nephrin itself, several humoral mediators are mechanistically implicated in the progression of hypertensive nephropathy. Transforming growth factor-*β*1 (TGF-*β*1) is the principal profibrotic cytokine driving glomerulosclerosis and tubulointerstitial fibrosis, with both canonical Smad-dependent and non-canonical signalling pathways now well characterised (22, 23). Aldosterone exerts direct profibrotic and pro-apoptotic effects on podocytes through mineralocorticoid receptor signalling, oxidative stress, and Sgk1 activation, and downregulates nephrin and podocin gene expression in experimental models (24, 25). Vascular endothelial growth factor-A (VEGF-A) reflects podocyte–endothelial crosstalk; both insufficient and excess VEGF-A signalling produce characteristic glomerular pathology, and elevated plasma VEGF-A has been validated as a marker of early vascular damage in hypertension (26, 27). The interrelationship between urinary nephrin and these circulating markers across hypertension stages, and their behaviour during standard antihypertensive and nephroprotective therapy with RAAS blockade 28, 29, has not been systematically characterised in the regional patient population. Against this background, we conducted a single-centre, stage-stratified study of 60 adults with essential hypertension in Bukhara, Uzbekistan. The aims of the study were threefold: (1) to characterise urinary nephrin concentrations across the clinical stages of essential hypertension and compare them with conventional renal markers; (2) to evaluate the relationship between urinary nephrin and serum profibrotic, RAAS, and angiogenic markers as well as renal hemodynamic indices and renal functional reserve; and (3) to assess the response of urinary nephrin to 6 months of standard antihypertensive therapy with an ACE inhibitor or angiotensin receptor blocker. We hypothesised that urinary nephrin would be elevated even in early-stage hypertension when conventional markers remain unremarkable, would correlate with markers of podocyte and endothelial injury, and would decline measurably under RAAS blockade.

## 2 Materials and methods

### 2.1 Study design and setting

This was a single-centre, prospective observational study with a 6-month follow-up component, conducted at the Bukhara Regional Multidisciplinary Hospital, Republic of Uzbekistan. The study was performed in accordance with the Declaration of Helsinki and approved by the Ethics Committee of the Bukhara State Medical Institute. Written informed consent was obtained from all participants prior to enrolment.

### 2.2 Study population

A total of 60 patients with established essential hypertension were consecutively enrolled. Hypertension was defined and staged according to the 2023 European Society of Hypertension Guidelines (29). Participants were stratified by hypertension stage into three groups of 20 patients each: stage I (mild, no target-organ damage), stage II (moderate, with target-organ involvement), and stage III (severe, with established cardiovascular or renal complications).

#### 2.2.1 Inclusion criteria

were: age 30–65 years; documented diagnosis of essential hypertension confirmed by office blood pressure ≥140/90 mmHg on at least two separate visits; absence of secondary causes of hypertension; and willingness to provide written informed consent and adhere to the 6-month follow-up protocol.

#### 2.2.2 Exclusion criteria

were: type 1 or type 2 diabetes mellitus; chronic heart failure (New York Heart Association class II or higher); known primary kidney disease; active malignancy; pregnancy or lactation; severe hepatic impairment; active infection or inflammatory disease at enrolment; and any prior or concurrent SARS-CoV-2 infection confirmed by PCR or serology, in order to isolate the effects of hypertension on podocyte function from those of post-viral renal injury. Diabetes mellitus was excluded at enrolment on the basis of a known clinical diagnosis or documented history of diabetes together with glycated haemoglobin (HbA1c) and fasting plasma glucose measured at screening; an oral glucose tolerance test was not performed. All enrolled patients had HbA1c below the diabetic threshold (cohort mean 5.2%, range 4.1%–5.8%; all < 6.5%) and fasting plasma glucose within the non-diabetic range across all stages, confirming the absence of diabetes.

### 2.3 Clinical assessment

At baseline and after 6 months of treatment, all participants underwent a standardised clinical evaluation including demographic data, disease duration, anthropometric measurements, and office blood pressure measurement performed in triplicate after 5 min of rest using a calibrated automated oscillometric device, with the average of the second and third readings recorded.

### 2.4 Laboratory measurements

Venous blood and morning mid-stream urine samples were collected after an overnight fast. Routine biochemistry was performed on an automated analyser using standard enzymatic and colorimetric methods. eGFR was calculated using the 2021 race-free CKD-EPI creatinine equation (9). Specialised renal biomarkers — urinary nephrin, serum cystatin-C, TGF-*β*1, VEGF-A, and aldosterone — were quantified by commercial enzyme-linked immunosorbent assay (ELISA) kits according to the manufacturers' protocols. The following kits were used: urinary nephrin (DiMediTec Diagnostics, Germany); serum aldosterone (DBC Aldosterone ELISA, Canada; assay range 15–2000ng/mL, sensitivity 15 ng/mL); and TGF-*β*1 and VEGF-A (quantitative sandwich ELISA). Serum cystatin-C was measured by an immunoturbidimetric assay (DiaSys, Germany; reference range 0.58–1.02 mg/mL), and routine biochemistry, including serum creatinine (Jaffe method), was performed on a Mindray BA-88 analyser. Microalbuminuria was determined in a 24-hour urine collection using an immunoturbidimetric assay. All assays were performed in duplicate. Urinary nephrin was measured in first-morning, mid-stream specimens, which reduces diurnal and hydration-related variability in urinary concentration. Urinary creatinine was not measured concurrently; urinary nephrin is therefore reported as an absolute concentration (ng/mL), and the potential influence of urine concentration on the reported values is addressed in the Limitations.

### 2.5 Renal functional reserve

Renal functional reserve (RFR) was assessed using a standardised oral protein-loading test, in line with established protocols (30, 31). After baseline measurement of eGFR under normal hydration, participants ingested an oral protein load of unsalted egg white (1 g per kg body weight) over 10–15 min between 08:00 and 08:30, and GFR was re-measured 1.5 h later. GFR before and after protein loading was derived from serum cystatin-C. RFR was expressed as the percentage increase in GFR from baseline to post-load values, and was categorised as preserved (increase > 10%), reduced (5%–10%), or absent (≤ 5%).

### 2.6 Imaging

All participants underwent transthoracic echocardiography and renal artery Doppler ultrasonography on the same day. Renal Doppler ultrasonography assessed three segments of the renal vasculature — the main, segmental, and interlobar arteries — bilaterally. Peak systolic velocity (Vps), end-diastolic velocity (Ved), resistive index (RI), and pulsatility index (PI) were recorded as the average of three consecutive measurements per vessel, in accordance with standard methodology for renal Doppler evaluation in hypertensive patients (32, 33).

### 2.7 Treatment protocol

Following baseline assessment, all participants received a standardised antihypertensive regimen for 6 months, based on the 2023 ESH guideline recommendations (29). First-line therapy consisted of either the ACE inhibitor enalapril (5 mg twice daily, up-titrated to 10 mg twice daily) or the angiotensin receptor blocker azilsartan (40 mg once daily, up-titrated to 80 mg). The mineralocorticoid receptor antagonist eplerenone (25–50 mg/day) was added in selected patients with persistently elevated serum aldosterone and inadequate blood pressure control, in line with guideline recommendations for resistant hypertension (29).

### 2.8 Statistical analysis

Continuous variables are presented as mean ± standard deviation (SD). The normality of distributions was assessed using the Shapiro–Wilk test. Between-group comparisons across the three hypertension stages were performed using one-way analysis of variance (ANOVA) with Tukey's honestly significant difference (HSD) test for *post-hoc* pairwise comparisons; non-normally distributed variables (fasting glucose) were compared using the Kruskal–Wallis test. Within-group pre- and post-treatment comparisons used the paired Student's t-test. Associations between urinary nephrin and other parameters were evaluated using Pearson correlation coefficients (Spearman where appropriate), with correction for multiple comparisons by the Benjamini–Hochberg false-discovery-rate procedure. To determine whether the association between urinary nephrin and renal function was independent of disease severity and age, a multivariable linear regression model was constructed with urinary nephrin as the dependent variable and age, systolic blood pressure, and eGFR as independent variables; standardised *β* coefficients with 95% confidence intervals are reported, and multicollinearity was evaluated using the variance inflation factor (VIF). A two-sided *P* < 0.05 was considered statistically significant. Analyses were performed using Python 3 (SciPy and statsmodels packages).

## 3 Results

### 3.1 baseline characteristics of the study cohort

A total of 60 patients with essential hypertension were enrolled and distributed equally across three groups by disease stage (*n* = 20 per stage). Mean age was 46.0 ± 7.3 years in stage I, 44.5 ± 8.9 years in stage II, and 52.3 ± 8.0 years in stage III, with a female-to-male ratio of approximately 2:1 across the cohort, consistent with the regional epidemiology of hypertension among middle-aged adults in Bukhara region.

Baseline clinical and laboratory parameters are summarised in Table 1. Systolic blood pressure was higher in stages II and III than in stage I (149.6 ± 7.6, 165.3 ± 10.4, and 164.2 ± 10.4 mmHg in stages I, II, and III, respectively). Conventional markers of renal function showed a stepwise deterioration: serum creatinine increased from 74.5 ± 5.4 µmol/L in stage I to 97.3 ± 5.2 µmol/L in stage III, serum cystatin-C from 0.8 ± 0.03 mg/mL to 1.1 ± 0.04 mg/mL, and estimated glomerular filtration rate (eGFR) decreased from 104 ± 6.2 to 72 ± 4.3 mL/min/1.73 m^2^. Importantly, in stage I patients, all three conventional markers remained within the upper limit of normal reference ranges, illustrating their limited sensitivity for early hypertensive kidney injury.

**Table 1:** Baseline clinical and laboratory characteristics of hypertensive patients across disease stages.

Parameter	Stage I (*n* = 20)	Stage II (*n* = 20)	Stage III (*n* = 20)
Age, years	46.0 ± 7.3	44.5 ± 8.9	52.3 ± 8.0
SBP, mmHg	149.6 ± 7.6	165.3 ± 10.4	164.2 ± 10.4
DBP, mmHg	90.8 ± 4.2	95.3 ± 5.6	100.2 ± 6.4
Glucose, mmol/L	3.7 ± 0.7	3.7 ± 0.7	4.8 ± 0.7
Total cholesterol, mmol/L	4.6 ± 1.1	4.8 ± 1.5	5.6 ± 0.6
Serum creatinine, µmol/L	74.5 ± 5.4	84.8 ± 4.2	97.3 ± 5.2
Cystatin-C, mg/mL	0.8 ± 0.03	1.0 ± 0.04	1.1 ± 0.04
eGFR, mL/min/1.73 m^2^	104 ± 6.2	89 ± 5.6	72 ± 4.3
Renal functional reserve, %	22.5 ± 3.1	15.5 ± 1.6	12.5 ± 1.6

SBP, systolic blood pressure; DBP, diastolic blood pressure; eGFR, estimated glomerular filtration rate (CKD-EPI 2021). Values are mean ± standard deviation (SD).

### 3.2 Urinary nephrin and associated profibrotic markers

**Figure 1:**
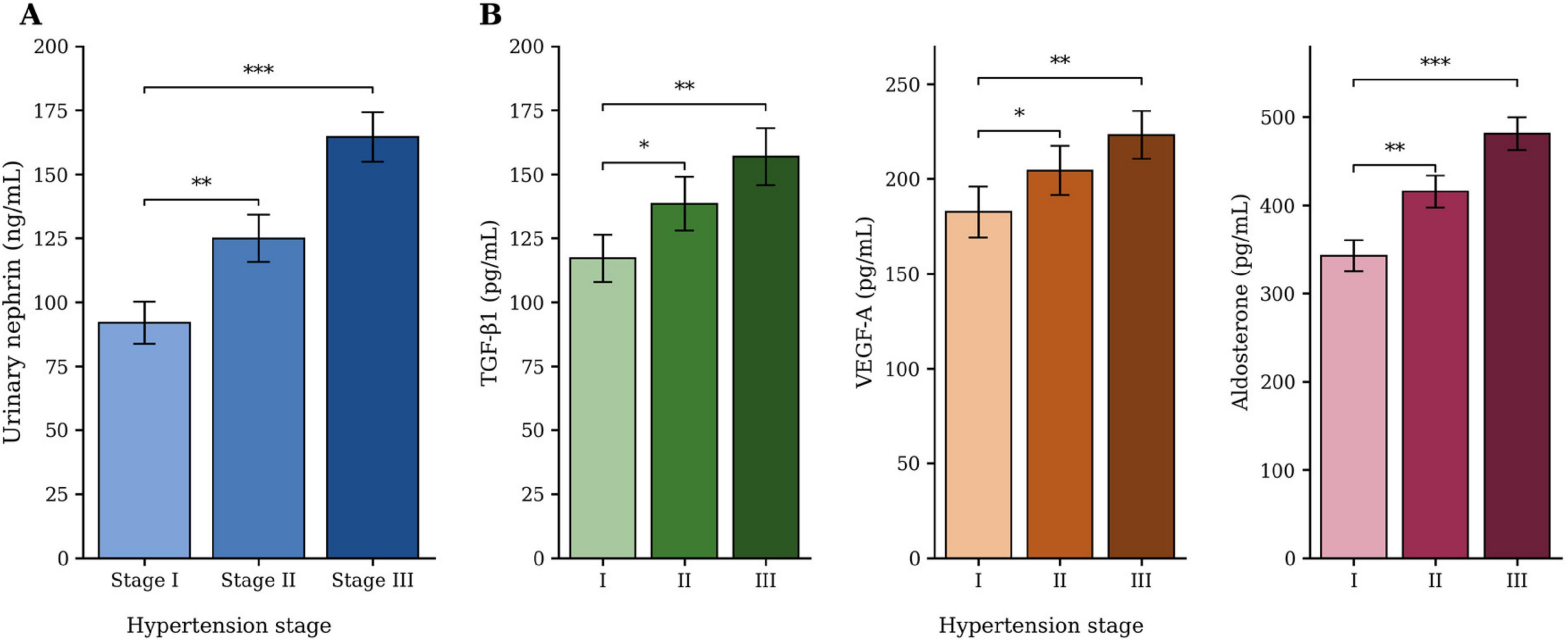
Biomarker profile across hypertension stages. **(A)** Urinary nephrin concentration (ng/mL) in patients with stage I, II, and III essential hypertension. **(B)** Serum levels of transforming growth factor-*β*1 (TGF-*β*1), vascular endothelial growth factor-A (VEGF-A), and aldosterone (pg/mL) across stages. Values are mean ± standard deviation (SD). *P < 0.05, **P < 0.01, ***P < 0.001 vs. stage I.

Urinary nephrin concentration demonstrated a clear stepwise increase across hypertension stages: 91.9 ± 8.3 ng/mL in stage I, 124.9 ± 9.3 ng/mL in stage II, and 164.5 ± 9.7 ng/mL in stage III (Figure 1A). Notably, nephrinuria was already markedly elevated in stage I patients, when serum creatinine, cystatin-C, and eGFR all remained within the reference range, supporting the hypothesis that podocyte-derived urinary nephrin reflects subclinical glomerular injury that is not yet reflected by global renal function.

Profibrotic and angiogenic markers showed a parallel pattern (Figure 1B). Serum TGF-*β*1 increased from 117.1 ± 9.3 pg/mL in stage I to 138.4 ± 10.4 pg/mL in stage II, with further elevation in stage III. Serum VEGF-A rose from 182.5 ± 13.5 pg/mL in stage I to 223.2 ± 12.6 pg/mL in stage III. Aldosterone concentrations increased markedly with disease severity, from 342.6 ± 17.5 pg/mL in stage I to 480.8 ± 18.6 pg/mL in stage III. The simultaneous elevation of urinary nephrin, TGF-*β*1, VEGF-A, and aldosterone across stages is consistent with a coordinated activation of podocyte injury, profibrotic signalling, and the renin-angiotensin-aldosterone system from early stages of essential hypertension.

### 3.3 Renal hemodynamics and functional reserve

Doppler ultrasonography of the renal arteries revealed progressive hemodynamic alterations across hypertension stages (Table 2). In the segmental arteries, peak systolic velocity (Vps) declined from 63.2 ± 3.2 cm/s in stage I to 44.5 ± 2.3 cm/s in stage III, while the resistive index (RI) rose from 0.58 ± 0.03 to 0.60 ± 0.03 and the pulsatility index (PI) increased from 1.04 ± 0.02 to 1.12 ± 0.03. Similar but more pronounced changes were observed in the interlobar arteries, where Vps decreased from 36.2 ± 1.8 to 30.5 ± 1.3 cm/s and PI rose from 0.88 ± 0.01 to 1.04 ± 0.03 (*P* < 0.05 for stage I vs. stage III).

**Table 2:** Renal artery Doppler parameters at baseline.

Vessel/Index	Stage I (*n* = 20)	Stage II (*n* = 20)	Stage III (*n* = 20)
Main renal artery			
Vps, cm/s	75.6 ± 3.3	70.6 ± 4.1	61.4 ± 3.4
Ved, cm/s	28.3 ± 2.8	22.5 ± 1.2	20.0 ± 2.2
RI	0.62 ± 0.02	0.65 ± 0.02	0.69 ± 0.02
PI	1.16 ± 0.02	1.22 ± 0.02	1.26 ± 0.02
Segmental artery			
Vps, cm/s	63.2 ± 3.2	50.2 ± 1.8	44.5 ± 2.3
Ved, cm/s	19.7 ± 1.3	18.8 ± 1.3	17.9 ± 1.1
RI	0.58 ± 0.03	0.59 ± 0.02	0.60 ± 0.03
PI	1.04 ± 0.02	1.10 ± 0.02	1.12 ± 0.03
Interlobar artery			
Vps, cm/s	36.2 ± 1.8	34.6 ± 1.6	30.5 ± 1.3
Ved, cm/s	18.2 ± 1.3	15.8 ± 1.0	14.6 ± 0.9
RI	0.56 ± 0.02	0.58 ± 0.02	0.60 ± 0.03
PI	0.88 ± 0.01	0.98 ± 0.03	1.04 ± 0.03

Vps, peak systolic velocity; Ved, end-diastolic velocity; RI, resistive index; PI, pulsatility index. Values are mean ± standard deviation (SD).

Renal functional reserve (RFR), assessed by a standardised protein-loading test, declined sharply with advancing disease: 22.5 ± 3.1% in stage I, 15.5 ± 1.6% in stage II, and 12.5 ± 1.6% in stage III (*P* < 0.001 across stages). Even in stage I, RFR values were already approaching the lower limit of the normal range, indicating reduced adaptive capacity of the kidneys before serum-based markers became overtly abnormal.

### 3.4 Associations between urinary nephrin and clinical parameters

Across the cohort, urinary nephrin concentration showed strong positive correlations with disease duration (r = 0.51; *P* < 0.01), systolic blood pressure (r = 0.58; *P* < 0.01), serum aldosterone (r = 0.54; *P* < 0.01), and VEGF-A (r = 0.49; *P* < 0.01). A strong positive association was also observed between urinary nephrin and microalbuminuria (r = 0.71; *P* < 0.001), supporting their complementary roles in detecting early renal injury. Substantial negative correlations were identified between nephrinuria and both eGFR (r = –0.74; *P* < 0.001) and RFR (r = –0.76; *P* < 0.001), indicating that higher urinary nephrin excretion is closely linked to declining global and reserve renal function. Critically, these associations were already detectable in stage I patients, suggesting that nephrinuria may flag early functional compromise that is not captured by routine serum creatinine measurement. In a multivariable linear regression model (urinary nephrin as the dependent variable; age, systolic blood pressure, and eGFR as predictors; R^2^ = 0.88), both systolic blood pressure (standardised *β* = 0.46; 95% CI 0.37–0.56; *P* < 0.001) and eGFR (standardised *β* = −0.68; 95% CI −0.78 to −0.58; *P* < 0.001) were independently associated with urinary nephrin, whereas age was not (*β* = 0.09; *P* = 0.07); all variance inflation factors were below 1.2, indicating no meaningful collinearity. This indicates that the inverse relationship between nephrinuria and renal function is independent of blood pressure and age. Across all biomarkers, one-way ANOVA was highly significant (all *P* < 0.001), and Tukey HSD *post-hoc* testing confirmed that every pairwise difference between stages I, II, and III was statistically significant (*P* < 0.001), including for urinary nephrin, eGFR, RFR, and aldosterone.

### 3.5 Response to 6-month antihypertensive and nephroprotective therapy

**Figure 2:**
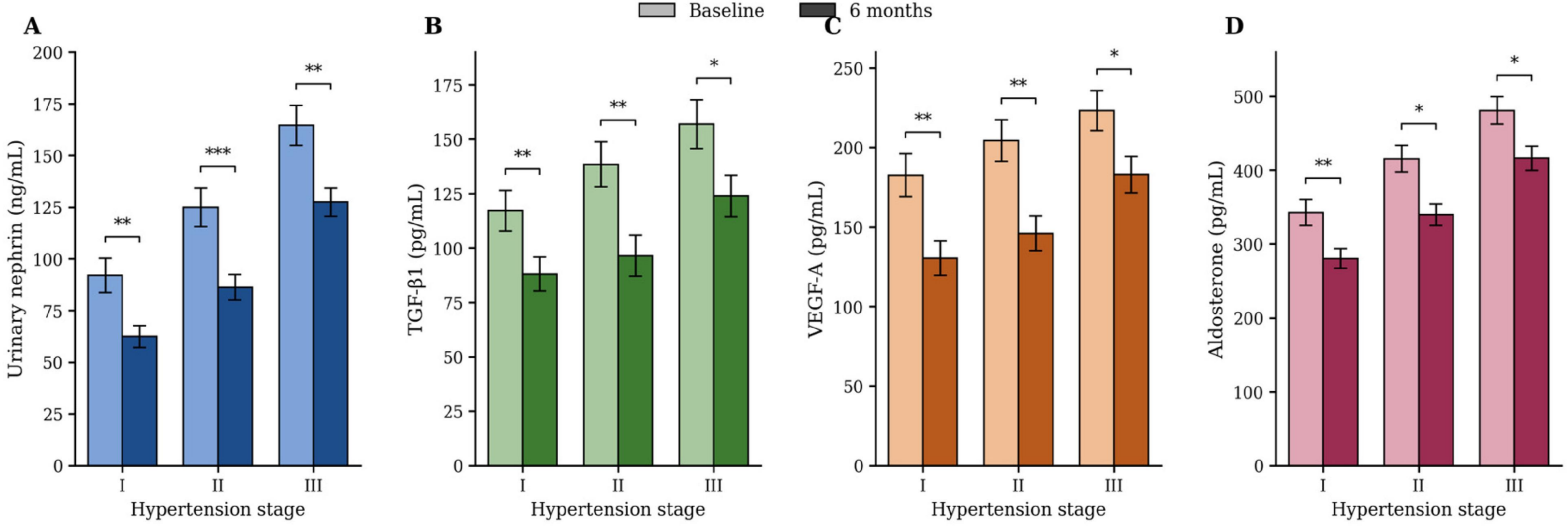
Effect of 6-month renin-angiotensin-aldosterone system blockade on biomarkers across hypertension stages. Pre- and post-treatment values of **(A)** urinary nephrin, **(B)** transforming growth factor-*β*1 (TGF-*β*1), **(C)** vascular endothelial growth factor-A (VEGF-A), and **(D)** aldosterone after 6 months of therapy with an ACE inhibitor (enalapril) or angiotensin receptor blocker (azilsartan), supplemented by eplerenone where indicated. Values are mean ± standard deviation (SD). *P < 0.05, **P < 0.01, ***P < 0.001 vs. baseline.

After 6 months of standard therapy with an ACE inhibitor (enalapril 5–10 mg) or ARB (azilsartan 40–80 mg), supplemented by eplerenone in cases of inadequate blood pressure control, significant biochemical and hemodynamic improvements were observed across all stages. Systolic blood pressure decreased from 149.6 ± 7.6 to 120.4 ± 5.4 mmHg in stage I (–21%; *P* < 0.01) and from 165.3 ± 10.4 to 126.6 ± 7.6 mmHg in stage II (–23%; *P* < 0.01). Urinary nephrin concentration declined substantially in all groups (Figure 2): from 91.9 ± 8.3 to 62.4 ± 5.3 ng/mL in stage I (–32%; *P* < 0.01), by 31% in stage II (*P* < 0.001), and from 164.5 ± 9.7 to 127.4 ± 6.8 ng/mL in stage III (–23%; *P* < 0.01). Serum TGF-*β*1 fell 1.4-fold in stage II (from 138.4 ± 10.4 to 96.4 ± 9.4 pg/mL; *P* < 0.01) and by 21% in stage III (*P* < 0.05). VEGF-A decreased 1.37–1.40-fold in stages I and II (*P* < 0.01) and by 18% in stage III (*P* < 0.05). Aldosterone declined from 342.6 ± 17.5 to 280.3 ± 13.2 pg/mL in stage I (*P* < 0.01) and from 480.8 ± 18.6 to 416.2 ± 16.4 pg/mL in stage III (*P* < 0.05). Renal Doppler parameters also improved: in main renal arteries, Vps increased by 22% (*P* < 0.01) and PI decreased by 10% (*P* < 0.05). Importantly, the reduction in nephrinuria after treatment was paralleled by a recovery of renal functional reserve, indicating that early nephroprotective intervention can attenuate proteinuric markers of podocyte injury and partially restore the adaptive renal capacity of hypertensive kidneys.

## 4 Discussion

### 4.1 Principal findings

In this single-centre, stage-stratified study of 60 patients with essential hypertension, three principal findings emerged. Urinary nephrin was already elevated in stage I disease and rose progressively across stages, while conventional markers remained within or near the reference range. Nephrinuria correlated strongly with markers of profibrotic activation (TGF-*β*1, aldosterone), endothelial dysfunction (VEGF-A), and renal hemodynamic deterioration. Six months of RAAS blockade produced a significant reduction in urinary nephrin paralleled by partial recovery of RFR.

### 4.2 Nephrinuria is elevated in the presence of normal conventional markers of renal injury

The dissociation between nephrinuria and standard renal indices in stage I patients of our cohort echoes findings from earlier studies demonstrating that urinary podocyte-associated proteins increase before albuminuria and detectable filtration loss (14, 17, 18). Wang and colleagues first showed that urinary nephrin is barely detectable in healthy individuals and chronic hypertension without proteinuria but rises sharply in pre-eclampsia and other podocytopathies (17). Subsequent work confirmed that podocyturia and nephrin shedding occur in the early course of human hypertensive renal injury, before substantial decline in eGFR (14). Our data extend these observations to stage I essential hypertension, where conventional markers including the 2021 CKD-EPI eGFR and serum cystatin-C remain unremarkable. The clinical relevance is reinforced by recent evidence that microalbuminuria is a relatively late event with limited sensitivity (around 44%) for early hypertensive nephropathy, leaving a substantial diagnostic gap that nephrinuria may help fill (10, 11).

### 4.3 Coordinated activation of profibrotic and RAAS pathways

The parallel rise in urinary nephrin, TGF-*β*1, VEGF-A, and aldosterone across hypertension stages observed in our cohort is mechanistically coherent. TGF-*β*1 is a master profibrotic cytokine that drives mesangial matrix expansion and tubulointerstitial fibrosis through canonical Smad2/3 and non-canonical pathways, and is now recognised as the principal mediator of CKD progression of varied aetiology (22, 23). Aldosterone exerts direct profibrotic effects through mineralocorticoid receptor activation in podocytes; experimental work by Shibata and colleagues demonstrated that aldosterone induces oxidative stress, downregulates nephrin and podocin gene expression, and produces overt podocyte injury, all of which are reversed by eplerenone (24, 25). The strong positive correlation between nephrinuria and aldosterone (r = 0.54) in our cohort is consistent with this mechanism and supports the therapeutic addition of mineralocorticoid receptor antagonists in selected patients.

The behaviour of VEGF-A in our data warrants nuanced interpretation. VEGF-A is essential for normal podocyte–endothelial crosstalk, but excess VEGF-A signalling in the hypertensive glomerulus has been implicated in maladaptive vascular remodelling (26). Belgore and colleagues demonstrated that plasma VEGF-A is elevated in untreated hypertensive patients and correlates with endothelial dysfunction even before overt vascular damage (27). Our finding of progressively rising serum VEGF-A across stages — together with worsening Doppler indices — supports the interpretation that VEGF-A elevation in this context reflects endothelial dysfunction rather than a protective response. This interpretation should, however, be qualified. VEGF-A exerts dual, context-dependent effects in glomerular biology: physiological podocyte-derived VEGF-A is indispensable for maintaining endothelial fenestration and filtration-barrier integrity, whereas both deficient and excessive signalling produce distinct glomerular pathology, giving a U-shaped dose–response relationship (26). Importantly, we measured circulating (systemic) VEGF-A, which integrates endothelial activation throughout the vasculature and need not parallel local intrarenal VEGF-A bioavailability or podocyte VEGF-A expression. Our results should therefore be read as describing an association between a systemic vascular marker and hypertension stage, rather than as a direct readout of intrarenal VEGF-A signalling; compartment-specific conclusions would require paired measurement of urinary or tissue VEGF-A.

### 4.4 Hemodynamic and functional consequences

Doppler indices in our cohort demonstrated a stepwise increase in resistive and pulsatility indices alongside falling peak systolic velocities, particularly in segmental and interlobar arteries — a pattern that reproduces the small-vessel intrarenal stiffening described in earlier hypertensive cohorts (32, 33). The renal resistive index has been validated as a useful tool not only for primary hypertension management but also as a predictor of CKD progression (32). The strong inverse correlation between nephrinuria and RFR (r = –0.76) is, in our view, the most clinically actionable finding of this study: it links a urinary biomarker that can be measured non-invasively in primary care to a functional parameter that quantifies adaptive renal capacity. RFR, first described by Bosch and colleagues and further refined for hypertensive populations (30, 31), is reduced before resting eGFR falls; patients identified by elevated nephrinuria therefore represent a target population in whom intensified blood pressure control and RAAS blockade may yield disproportionate long-term benefit.

### 4.5 Therapeutic responsiveness of nephrinuria

The 23%–32% reduction in urinary nephrin observed across stages after six months of ACE inhibitor or ARB therapy is consistent with the established nephroprotective effects of RAAS blockade, which lower intraglomerular pressure, reduce hyperfiltration, and attenuate proteinuria independently of systemic blood pressure reduction (28, 29). The magnitude of nephrinuria reduction was greatest in stage I patients (–32%), supporting earlier intervention. The parallel improvement in RFR suggests that the reduction in nephrin shedding is not merely a laboratory phenomenon but reflects genuine restoration of glomerular filtration barrier integrity. These observations argue for incorporating urinary nephrin into therapeutic monitoring protocols, particularly in patients with normal eGFR but elevated baseline nephrinuria.

### 4.6 Limitations

Several limitations should be acknowledged. First, this is a single-centre study with a relatively small sample (*n* = 60), and the findings require confirmation in larger, multi-centre cohorts. Second, we did not include an age- and sex-matched normotensive control group; consequently, the characterisation of stage I nephrin values as “elevated” is necessarily relative to the higher hypertension stages and to published reference ranges, and absolute thresholds for normality in this population cannot be derived from the present data. Third, the design was observational and cross-sectional with only six months of follow-up; although a multivariable model supported an independent association between nephrinuria and renal function, the design does not establish temporal precedence, and the findings should be regarded as hypothesis-generating rather than as evidence that nephrinuria rises before conventional markers in any given patient. Fourth, treatment was not randomised and lacked a placebo arm (ethically precluded in established hypertension); patients receiving an ACE inhibitor, an angiotensin receptor blocker, or eplerenone were pooled, and treatment-subgroup allocation and baseline comparability were not formally analysed, so the observed biomarker changes should be attributed to renin-angiotensin-aldosterone system blockade as a class rather than to any individual agent. Fifth, urinary nephrin was reported as an absolute concentration without normalisation to urinary creatinine, which was not measured concurrently; although first-morning, mid-stream specimens were used to limit hydration-related variability, residual variation in urine concentration may have influenced the reported values. Sixth, patients with prior SARS-CoV-2 infection were excluded to isolate hypertensive podocyte injury, which strengthens internal validity but may limit generalisability. Finally, inter-laboratory standardisation of nephrin ELISA remains an unresolved challenge, and emerging mass-spectrometry approaches may provide more reproducible measurements in future (19).

### 4.7 Implications and future directions

Despite these limitations, our findings have practical implications for hypertension management in resource-constrained settings such as Central Asia, where access to renal biopsy and advanced imaging is limited. Urinary nephrin is technically straightforward to measure, requires only a spot urine sample, and shows excellent dynamic range across hypertension stages. Prospective validation studies should focus on (1) defining clinically meaningful cut-off values for stage-specific risk stratification, (2) determining whether nephrinuria-guided intensification of RAAS blockade improves long-term renal outcomes, and (3) clarifying the relationship between nephrinuria and incident cardiovascular events. Until such data become available, our results support the use of urinary nephrin as a complementary tool — alongside microalbuminuria and eGFR — for identifying hypertensive patients at elevated risk of progressive nephron loss.

## Data Availability

The original contributions presented in the study are included in the article/Supplementary Material, further inquiries can be directed to the corresponding author.
